# In Vitro Susceptibility of Multi-Drug Resistant *Klebsiella* *pneumoniae* Strains Causing Nosocomial Infections to Fosfomycin. A Comparison of Determination Methods

**DOI:** 10.3390/pathogens10050512

**Published:** 2021-04-23

**Authors:** Beata Mączyńska, Justyna Paleczny, Monika Oleksy-Wawrzyniak, Irena Choroszy-Król, Marzenna Bartoszewicz

**Affiliations:** 1 Department of Pharmaceutical Microbiology and Parasitology, Faculty of Pharmacy, Medical University, 50-367 Wroclaw, Poland; paleczny.justyna@gmail.com (J.P.); monika.oleksy@umed.wroc.pl (M.O.-W.); m.bartoszewicz@op.pl (M.B.); 2 Department of Basic Sciences, Faculty of Health Sciences, Medical University, 50-367 Wroclaw, Poland; irena.choroszy-krol@umed.wroc.pl

**Keywords:** *Klebsiella pneumoniae*, fosfomycin, multi-drug resistance strains, resistance testing methods

## Abstract

Introduction: Over the past few decades, *Klebsiella pneumoniae* strains increased their pathogenicity and antibiotic resistance, thereby becoming a major therapeutic challenge. One of the few available therapeutic options seems to be intravenous fosfomycin. Unfortunately, the determination of sensitivity to fosfomycin performed in hospital laboratories can pose a significant problem. Therefore, the aim of the present research was to evaluate the activity of fosfomycin against clinical, multidrug-resistant *Klebsiella pneumoniae* strains isolated from nosocomial infections between 2011 and 2020, as well as to evaluate the methods routinely used in hospital laboratories to assess bacterial susceptibility to this antibiotic. Materials and Methods: 43 multidrug-resistant *Klebsiella* strains isolates from various infections were tested. All the strains had ESBL enzymes, and 20 also showed the presence of carbapenemases. Susceptibility was determined using the diffusion method (E-test) and the automated system (Phoenix), which were compared with the reference method (agar dilution). Results: For the reference method and for the E-test, the percentage of strains sensitive to fosfomycin was 65%. For the Phoenix system, the percentage of susceptible strains was slightly higher and stood at 72%. The percentage of fosfomycin-resistant strains in the *Klebsiella* carbapenemase-producing group was higher (45% for the reference method and E-test and 40% for the Phoenix method) than in carbapenemase-negative strains (25%, 25%, and 20%, respectively). Full (100%) susceptibility categorical agreement was achieved for the E-test and the reference method. Agreement between the automated Phoenix system and the reference method reached 86%. Conclusions: Fosfomycin appears to be the antibiotic with a potential for use in the treatment of infections with multidrug-resistant *Klebsiella* strains. Susceptibility to this drug is exhibited by some strains, which are resistant to colistin and carbapenems. The E-test, unlike the Phoenix method, can be an alternative to the reference method in the routine determination of fosfomycin susceptibility, as it shows agreement in terms of sensitivity categories and only slight differences in MIC values. The Phoenix system, in comparison to the reference method, shows large discrepancies in the MIC values and in the susceptibility category.

## 1. Introduction

Fosfomycin, a natural bactericidal antibiotic and a derivative of phosphoric acid, has been used worldwide for more than 40 years. Currently, there is a revival of its use in antibiotic therapy. It gained renewed interest due to an increase in infections caused by multidrug-resistant bacteria, where therapeutic options are very limited [1,2,3].

Fosfomycin is a bactericidal antibiotic whose unique properties are owed, among other things, to its mechanism of action. It is involved in inhibiting cell wall synthesis, but in a different way than in the case of β-lactam or glycopeptide antibiotics. It inhibits the MurA enzyme (UDP-*N*-acetylglucosamine-enolpyruvyltransferase), which is responsible for the synthesis of the pentapeptide mediating the synthesis of peptidoglycan (bacterial cell wall component). At the same time, i.v. fosfomycin, which has the smallest hydrophilic molecule among all antibiotics, has good pharmacokinetic properties, including excellent distribution to many tissues. This antibiotic achieves clinically relevant concentrations in the serum (does not bind with plasma proteins), kidneys, lungs, bones, heart valves, bladder, prostate gland, and seminal glands. It penetrates the placenta, as well as the blood-brain barrier, reaching high concentrations in the cerebrospinal fluid. It also demonstrates very good penetration into abscesses. It is nearly completely (95%) excreted unchanged in the urine within 24 h, and its half-life is relatively long (4–8 h). Due to its different chemical structure and characteristic mechanism of action, for the time being, there is no cross-resistance observed with other antibiotics commonly used in clinical practice [4,5,6]. Fosfomycin’s effective penetration into bacterial biofilm was also confirmed, in particular into the biofilm produced by the *Pseudomonas aeruginosa* and *Staphylococcus aureus* strains [7,8].

For many years, the use of fosfomycin was limited to the oral salt form with tromethamine (fosfomycin tromethamine), mainly in the treatment of uncomplicated urinary tract infections (UTI). Intravenous fosfomycin (disodium salt) was not registered by the European Commission until 2015 and initially was used in such EU countries as Spain, Germany, and France [9]. In Poland, on the other hand, i.v. fosfomycin was not registered until June 2019 [10].

Intravenous fosfomycin gained increased interest in the second decade of the 21st century, as it remains active against many very problematic pathogens such as methicillin-resistant *Staphylococcus aureus* (MRSA), *Enterococcus* strains resistant to glycopeptides [5], and multidrug–resistant (MDR) *Enterobacterales* [1,11,12,13]. Many publications also suggest potential activity of fosfomycin against *Pseudomonas aeruginosa,* noting however its low effectiveness when used in monotherapy [5,7,14]. The main indications for use are severe nosocomial infections such as complicated UTIs, cases of nosocomial pneumonia (including those associated with mechanical ventilation), meningitis, and sepsis occurring in the aftermath of any of the above infections. The therapeutic efficacy of fosfomycin is demonstrated by the fact that in many countries, the above indications were extended to include infections of bones and joints, complicated infections of the skin and soft tissues, complicated intra-abdominal infections, and infectious endocarditis [10].

Currently, clinicians and scientists are conducting a number of studies testing the susceptibility of different organisms to fosfomycin (by comparing diffusion and manual methods as well as automated dilution methods in liquid and solid media), hoping for its application in combination treatment of severe infections caused by multidrug-resistant pathogens that have very limited or no therapeutic options. However, there is a great need for modern research into the activity and range of action of fosfomycin, as well as into optimal methods for routine determination of in vitro resistance. 

Multidrug-resistant *Klebsiella pneumoniae* strains are among the pathogens posing a major therapeutic problem [1,3,4]. *Klebsiella* bacilli have undergone a unique evolution over the last few decades. They greatly increased their pathogenicity and reduced their susceptibility to antibiotics through acquiring new virulence features and resistance mechanisms. This has led to their transformation from bacteria commonly found in the environment, in the gastrointestinal (GI) tract, and in the upper respiratory tract of humans into dangerous pathogens with very high pathogenic potential and limited therapeutic options [15,16,17]. 

The greatest evolution took place in the production of broad-spectrum enzymes breaking down many classes of antibiotics, such as the ESBL enzymes. In the present decade, the strains often produce several types of ESBL, where on one plasmid ESBL, there are genes that inactivate other groups of antibiotics, and sensitivity remains only to carbapenems and colistin [1,17]. Currently, the greatest danger is posed by carbapenemase-producing strains, for which the therapeutic options are even more limited [18]

Drug-resistant *Klebsiella* strains currently pose a high risk, in both in-hospital and out-of-hospital settings, causing pneumonia; infections of wounds, the urinary tract, the central nervous system (CNS), and soft tissues; and septic infections (blood infections), which are particularly dangerous and have high mortality rates [3,16,17]. More and more frequently, we have to deal with strains which are multidrug-resistant (MDR), extensively drug-resistant (XDR), and even pandrug-resistant (PDR), i.e., strains resistant to almost all available drugs [1,18].

The proliferation of new antimicrobial resistance mechanisms in common pathogens is a growing concern and warrants the search for new therapeutic options. However, testing bacterial susceptibility to fosfomycin poses a certain problem because the reference method (serial dilutions of the drug in agar), which is the most reliable and recommended by EUCAST (European Committee on Antimicrobial Susceptibility Testing), is labor-intensive and difficult for routine use in hospital laboratories [19,20,21].

In view of the above facts, the main objective of this study was to evaluate the antimicrobial activity of fosfomycin against clinical multidrug-resistant strains of *Klebsiella pneumoniae* isolated from nosocomial infections over the past ten years. Another important goal was to evaluate the methods of determining bacterial susceptibility to fosfomycin routinely used in hospital laboratories: the diffusion methods (strips with concentration gradient, i.e., the E-test) and the automated method of serial dilutions in a liquid medium (i.e., the Phoenix system); the goal was to compare them with the method recommended by specialists—dilution in a solid medium. The use in microbiological laboratories of simpler and cheaper diffusion methods or the determination of fosfomycin susceptibility in automated systems raises doubts and is associated with the risk of obtaining false results, leading to incorrect antibiotic therapy of patients [20,21].

## 2. Results

All the test strains were identified by BD Phoenix as *Klebsiella pneumoniae.* The antibiograms obtained using the automated BD Phoenix system showed that the tested strains were highly resistant to routinely used antibiotics. All tested *Klebsiella* were characterized by complete resistance to aminopenicillins, penicillins with inhibitors of β-lactamases (excluding piperacillin/tazobactam—12% of sensitive strains), cephalosporins, quinolones, and, significantly, tigecycline and tobramycin. For the remaining aminoglycosides, the sensitivity of the tested *Klebsiella* isolates was higher (amikacin—66% sensitive strains, gentamycin—33%). The degree of sensitivity of the isolated bacilli to cotrimoxazole also varied, but resistant strains prevailed (86%).

**Figure 1:**
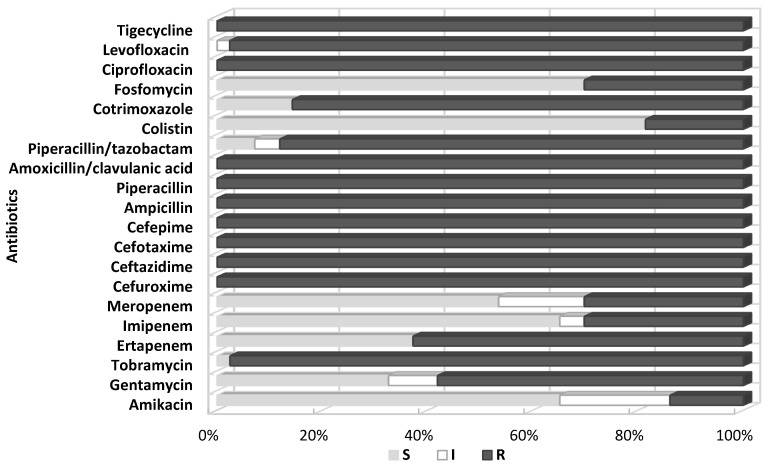
Percentage of tested *Klebsiella pneumoniae* strains susceptible and resistant to the selected antibiotics, determined by the Phoenix automated system; S—susceptible; I—susceptible, increased exposure; R—resistant.

Differences were noted in susceptibility to carbapenems (ertapenem, imipenem, meropenem), which correlated with the production by the tested strains of carbapenemases, although some carbapenemase-negative bacilli (six strains) also showed reduced susceptibility or resistance to these antibiotics, especially ertapenem. The tested strains showed the highest susceptibility to colistin (81% S), fosfomycin (71% S), imipeneme (65% S), and meropeneme (53% S). Six strains from a relatively small colistin-resistant group (19%) showed at the same time susceptibility to fosfomycin. Figure 1. shows a summary of susceptibility to individual antibiotics of all tested *Klebsiella pneumoniae* isolated at the clinical centers selected for the study.

**Figure 2:**
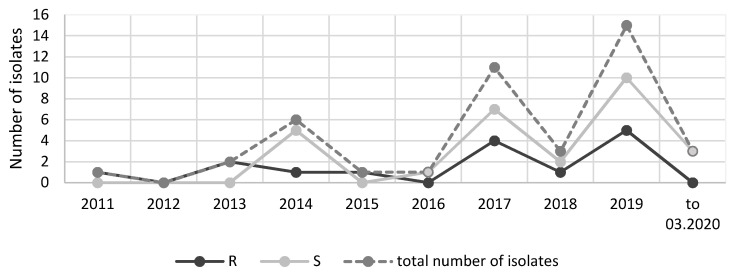
Comparison of the number of strains resistant and susceptible to fosfomycin isolated over the years; S—susceptible; R—resistant.

Over a period of five years (2011–2015), ten strains showing resistance to the majority of antibiotics used were collected for investigation. At that time, such multidrug-resistant *Klebsiella* strains were isolated rather infrequently at the hospitals selected for this study. In the next five years (2016–2020), as many as 33 of such multidrug-resistant strains were isolated. Most isolates were fosfomycin-susceptible strains. The comparisons conducted in individual years of the number of strains resistant to this antibiotic with the susceptible strains and with all isolated strains did not show any clear trend of increasing fosfomycin resistance in *Klebsiella* strains (Figure 2). The low number of isolates in 2020 is due to a short strain collection period (three months).

The presence of ESBL enzymes was confirmed in all strains. ESBL+ strains isolated in the study showed sensitivity to carbapenems, and resistance also to other groups of antibiotics, mainly fluoroquinolones, aminoglycosides, cotrimoxazole, and tigecycline. Twenty of them also showed the presence of carbapenemases (46.5%). MBL enzymes were detected in 16 of the tested *Klebsiella pneumoniae* strains (80%), and KPC enzymes were found in four strains (20%) (Supplementary Table S2). In the strain groups isolated in 2011–2015 and in 2016–2020, the number of carbapenemase-producing strains was similar (40% and 48%, respectively). The greatest number of strains produced MBL carbapenemases including VIM-type (one strain), NDM-1-type (14 strains), and 1 NDM-1/OXA (one strain).

**Figure 3:**
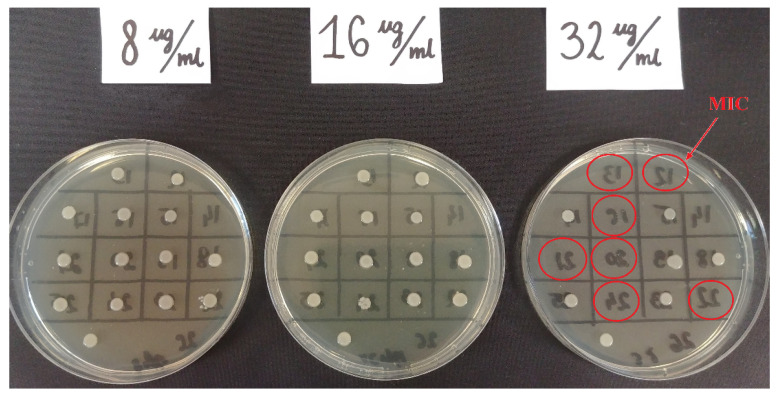
Example of fosfomycin MIC reading determined by the reference method for *Klebsiella pneumoniae* strains. The red circles indicates the MIC 32 mg/mL successively for strains no 12, 13, 16, 20, 21, 22, 24.

**Figure 4:**
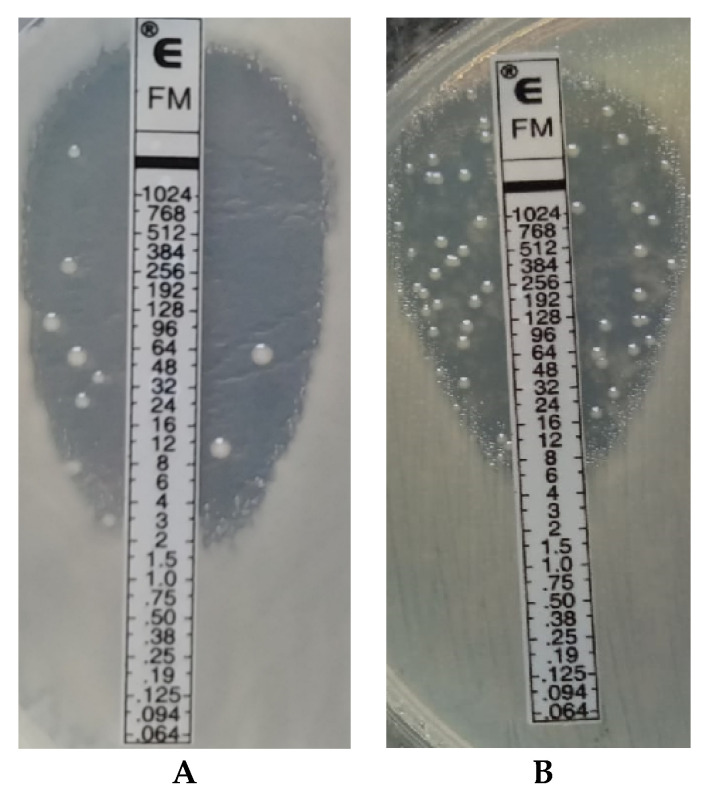
The E-Test method. Example of the reading of MIC for fosfomycin for *Klebsiella* strain no 5A (**A**); Example of the reading of MIC for fosfomycin for *Klebsiella* strain no 34D (**B**).

**Figure 5:**
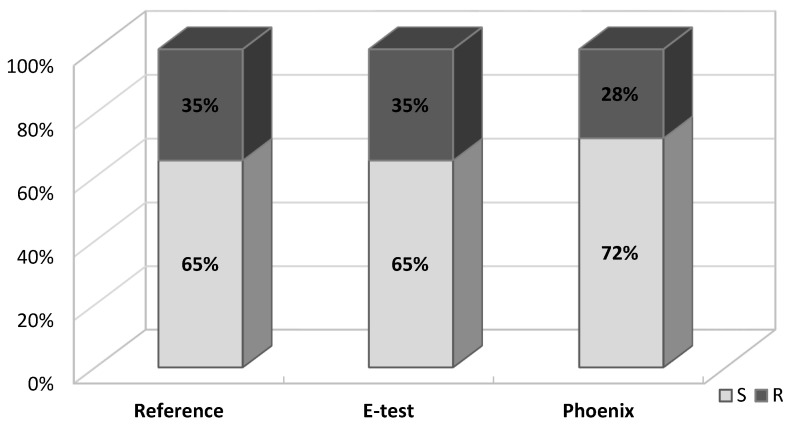
Percentage of fosfomycin-susceptible and fosfomycin-resistant *Klebsiella pneumoniae* strains detected by three methods: reference, E-test, automated Phoenix method; S—susceptible, R—resistant.

Fosfomycin sensitivity of multidrug-resistant *Klebsiella* strains was determined using three methods: the agar dilution method (reference method) (Figure 3), the gradient strip diffusion method (E-test) (Figure 4), and the automated method (Phoenix system). The results obtained by the diffusion and automated method were compared with the reference method. Figure 5 shows the percentage of fosfomycin-sensitive and fosfomycin-resistant strains determined by three methods. The strains tested were assigned to two categories: S—susceptible and R—resistant. 

**Figure 6:**
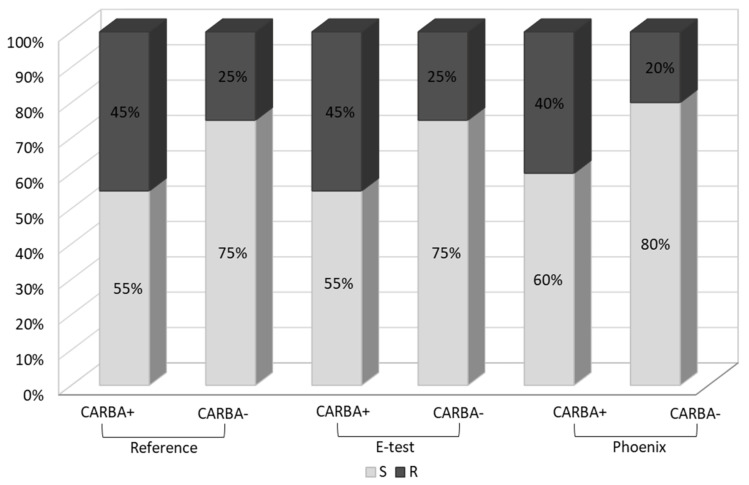
Percentage of fosfomycin-susceptible and fosfomycin-resistant strains in carbapenemase-positive and carbapenemase-negative *Klebsiella pneumoniae* strains, detected by three methods: reference, E-test and Phoenix; S—susceptible, R—resistant. As a positive control reference strain, *K. pneumoniae* ATCC BAA-1705 (KPC production) was used.

A similar analysis was carried out in the carbapenemase-producing group (CARBA+, *n* = 20) and in the group which does not produce this enzyme (CARBA−, *n* = 23). In the *Klebsiella* group of carbapenemase producers, the percentage of fosfomycin-resistant strains was significantly higher (45% using the reference and E-test methods and 40% using the Phoenix system) than in the group which does not produce carbapenemases (25%, 25% and 20%, respectively) (Figure 6).

### 2.1. Categorical Agreement

The E-test method and the reference method showed 100% agreement in terms of the susceptibility category. The agreement between the automated system and the reference method was 86%. Four VME (very major errors) and two 2 ME (major errors) were detected in the case of the Phoenix automated system. The VME rate and ME rate for the Phoenix system were 33% and 17%, respectively (Table 1).

**Figure 7:**
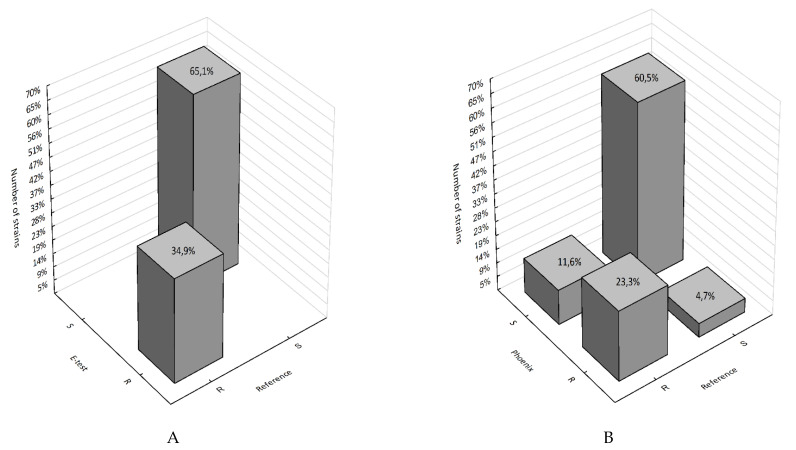
3D histograms of correlations between the number of fosfomycin-susceptible and fosfomycin-resistant strains determined by the E-test, Phoenix, and the reference method; (**A**) reference method vs. E-test; (**B**) reference vs. Phoenix; R—strain resistant to fosfomycin; S—strain susceptible to fosfomycin.

Figure 7, on the other hand, shows the percentage of falsely susceptible results (11.6%) and falsely resistant results (4.7%) obtained using the Phoenix system vs. the reference method (Figure 7B) and the absence of such results (100% categorical agreement) for the E-test method (Figure 7A).

A comparison of results obtained using the reference and E-test methods showed a statistically significant correlation (*p* < 0.000001). This is confirmed by the maximum value of phi coefficient Φ = 1, which indicates a perfect association between the reference method and the E-test. In the comparison between fosfomycin susceptibility results obtained using the reference method and the automated Phoenix system, the *p*-value is very low (*p* = 0.00008) and the phi coefficient Φ = 0.63, which indicates a correlation with a moderate correlation strength.

### 2.2. MIC Agreement

**Figure 8:**
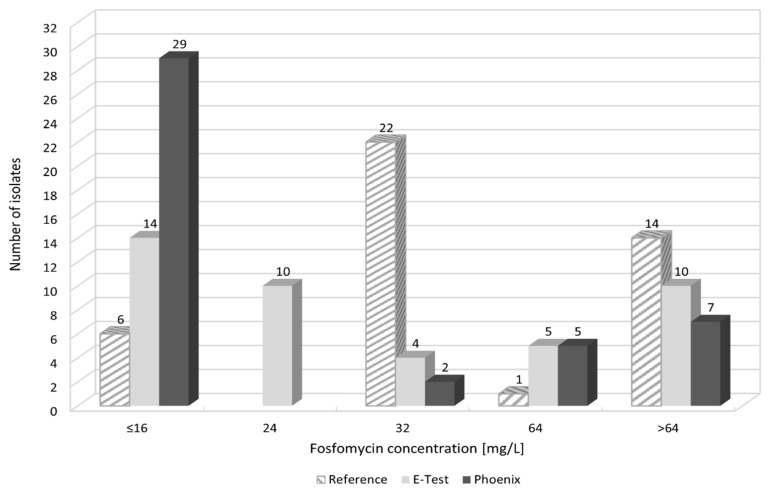
MIC distribution of fosfomycin of tested *Klebsiella pneumoniae* strains; because of the method’s use for the purpose of analysis, MIC ≤ 16 mg/L was substituted with 16 mg/L and >64 mg/L with 128 mg/L, >512 mg/L with 1024 mg/L; substitutions do not influence the diagnostic decision.

When comparing fosfomycin MIC values obtained using individual methods, differences were found in the ranges of values. Figure 8 and Figure S1A–C show the distribution of fosfomycin MIC values for each strain, obtained using the reference, E-test, and Phoenix methods.

**Figure 9:**
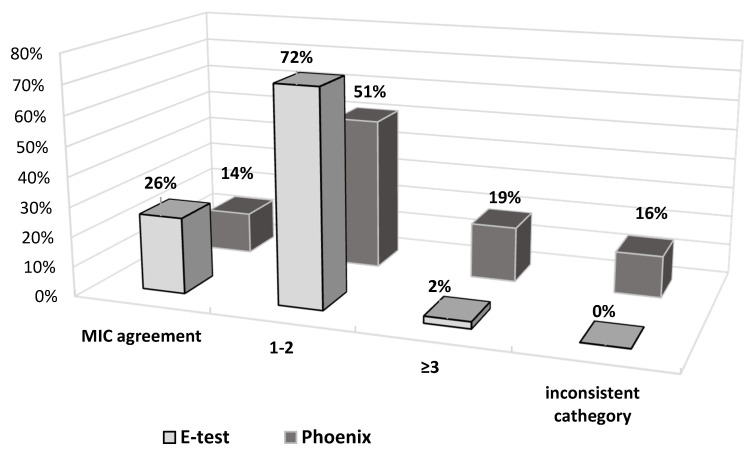
MIC deviations obtained between E-test and Phoenix methods compared to the reference method; 1-2—difference of one or two logarithmic dilutions, ≥3—difference of three or more logarithmic dilutions.

Figure 9 also shows the obtained differences in fosfomycin MIC values. In the E-test method, only 2% of results differed significantly (difference in three or more logarithmic dilutions) from the MIC values obtained by the reference method. A prevailing majority (98%) showed full agreement or a difference by one or two logarithmic dilutions (26% and 72%, respectively). This was not the case with the Phoenix automated method: as much as 33% of fosfomycin MIC values were completely inconsistent with the MIC values obtained in the reference method, resulting in a change in susceptibility category, or they differed significantly (14% and 19%, respectively).

A comparison of MIC values obtained in the E-test and the reference method showed a very high and statistically significant correlation (r = 0.83). Pearson’s correlation coefficient for MIC values obtained in the Phoenix method, and the reference method is r = 0.59, indicating a moderate correlation. 

The resistance analysis of the test strains in terms of site of infection (type of diagnostic material) did not show a statistically significant relationship between the site of isolation of the *Klebsiella pneumoniae* strain and its fosfomycin resistance (Table 2).

## 3. Discussion

*Klebsiella pneumoniae* is an important etiological factor in nosocomial and non-nosocomial infections, which are a serious threat to patients’ lives. For many years, these bacteria were a major challenge for clinicians around the world. Of particular concern is the fact that the organisms are able to survive and spread easily in hospital settings. An important role in the spread of the bacteria is played by gastrointestinal carriage, occurring in as much as 5–38% of the general population and in up to 77% of hospitalized patients [17,22]. The likelihood of colonization with multidrug-resistant strains increases with the duration of hospitalization [23]. According to the research by Lübbert et al., *Klebsiella pneumoniae* can be carried in the GI tract for one or even two years, despite previous assumptions of that period being three months [24].

The extremely rapid development of resistance mechanisms in these bacteria led to the elimination of almost all antibiotics from effective therapy [25]. Recently, new clones emerged capable of causing outbreaks and producing various types of extended-spectrum β-lactamases, including particularly dangerous carbapenemases [26]. Colonization of the gastrointestinal tract and upper respiratory tract by multidrug-resistant strains and the ability to transmit virulence-encoding genes facilitate the spread of pathogenic strains [17,22,23,24]. The epidemic nature of *Klebsiella* is illustrated in the statistics presented in the Annual Report of the European Antimicrobial Resistance Surveillance Network (EARS-Net) for most European countries [27]. The reports published in Poland also note a recent=year increase in the number of hospital outbreaks caused by *Klebsiella pneumoniae* [28]. Strains producing ESBL enzymes are currently common worldwide and, as was shown in this paper, beyond β-lactams, these organisms are resistant to many other antibiotics [29]. Today, carbapenemase-producing *Enterobacterales* (CPE) are among Europe’s most serious medical and epidemiological problems. The number and rate of the appearance of carbapenemase-producing strains indicate a high epidemic risk for hospital environments [30,31]. In the last decade (the test strains were isolated over the past ten years), Europe has witnessed an increasing spread of carbapenemase-producing *Klebsiella pneumoniae* strains. In the country of these authors, strains producing KPC enzymes (*Klebsiella Pneumoniae Carbapenemases*) appeared as the first ones, and their rapid spread was noted in 2010–2013. Since 2016, there was a marked increase in the occurrence of *Klebsiella* strains with NDM-1 carbapenemases *(New Delhi Metallo-β-lactamases*), and from 2017 this was also observed with OXA-48 enzymes (*Carbapenem-hydrolysing Class D β-lactamases*) [32,33,34]. In line with these data, in 2011–2015, in the multidrug-resistant isolates of *Klebsiella pneumoniae* studied in this paper, in addition to ESBL enzymes, mainly KPC carbapenemases were detected (Supplementary Table S2). Among the strains isolated in 2016–2020, NDM-1 carbapenebase-producing strains are predominant. However, the region of Poland, being the subject of this researchm has long been an area of rare occurrence of such strains. One of the most recently studied strains (March 2020), isolated from a female patient who returned from Turkey, produced as many as two carbapenemases (NDM-1 and OXA-48). Therapy of infections caused by carbapenemase-producing bacteria is extremely difficult; it requires coordinated interventions and combination treatment often including three or four drugs [35,36]. Unfortunately, according to the data from EARS-Net (European Antimicrobial Resistance Surveillance Network), since 2012, *Klebsiella* strains’ simultaneous resistance against cephalosporins, fluoroquinolones, and aminoglycosides remained above 50%, and since 2016, their resistance against colistin and carbapenems increased significantly. In the present research, we have observed, among others, strains sensitive to only one or two antibiotics (most commonly, fosfomycin or colistin).

This paper presents analyses of the sensitivity to fosfomycin of multidrug-resistant strains of *Klebsiella pneumoniae* isolated from four multi-specialty hospitals between 2011 and 2020. Like colistin, fosfomycin is a drug for which new applications have now been found [1,3,37,38]. Due to the almost complete lack of therapeutic options in some recently reported infections, medicine is returning to long-registered but relatively rarely used drugs, with new intensive research being conducted on them. The experiments carried out for the purposes of this paper aimed to assess the chances of effective therapy with fosfomycin of infections caused by multidrug-resistant *Klebsiella pneumoniae,* but also to verify the reliability of laboratory determinations of susceptibility to this drug.

The widespread and not always rational antibiotic therapy as well as *Enterobacterales’* ability for easy transmission of resistance genes significantly limited the options for effective antibacterial therapy. The above is confirmed in the review of antibiograms made for the tested pathogens using the automated Phoenix system (Figure 1). The analysis concerned bacteria with a high pathogenic potential. All tested *Klebsiella pneumoniae* strains produced ESBL β-lactamases and demonstrated resistance to cephalosporins of the second and third generation (except cephamycin) and to monobactams. As many as 47% of these strains produced carbapenemases hydrolysing antibiotics with β-lactamase inhibitors, other cephalosporins, and carbapenems (Supplementary Table S2). Additionally, development of resistance to quinolones, tigecycline, and cotrimoxazole was observed. A large part of the tested bacterial group did not show susceptibility to aminoglycosides. The acquisition by 19% of the analyzed *K. pneumoniae* of resistance to colistin raises concern, as colistin is often the only alternative in treating infections with carbapenemase-producing strains. The proportion of fosfomycin-susceptible strains detected with this method was as high as 72%, which classifies this antibiotic as the second most effective therapeutic option, alongside colistin in the treatment of severe infections with multidrug-resistant *Klebsiella* strains (Figure 1).

Due to the increasing therapeutic use of fosfomycin, it seems reasonable to analyze the trend of increasing resistance to this antibiotic. In light of the real risk of its rise, it is certainly necessary to monitor the resistance mechanisms and to track the spread of fosfomycinresistance genes [39]. The present study aimed to investigate the number of fosfomycin-resistant strains in relation to the number of susceptible strains over a period of ten years of their isolation. However, there was no clear trend towards increased resistance to this drug (Figure 2). However, because in certain years the number of analyzed strains was too small to allow fully reliable conclusions, the authors intend to continue their research in the future.

Thanks to the specific pharmacokinetic properties and the unique mechanism of action, i.v. fosfomycin can be successfully used in combination therapy. Except for urinary tract infections, monotherapy with i.v. fosfomycin is not recommended due to the risk of development of resistance [40,41]. A good safety profile of the agent allows for infusions in a wide dosage range. In addition, it is possible to combine fosfomycin with drugs that are potentially toxic such as colistin, aminoglycosides, or tigecycline. Synergism of action was also found between fosfomycin and cephalosporins [3,6,40]. An extensive literature presents i.v. fosfomycin as a viable treatment option for patients with systemic infections with *Enterobacterales* producing both ESBL and CPE [1,3,4,13,41,42]. As early as in 2010, Falagaset et al. reported the satisfactory sensitivity of multidrug-resistant *Klebsiella pneumoniae* to fosfomycin [1]. A review of research of *Enterobacteriaceae* with an advanced resistance profile and of their susceptibility to fosfomycin enabled the authors to collect data on a large group of *Klebsiella pneumoniae* strains. The research on *Klebsiella* strains not producing extended-spectrum β-lactamases shows that the percentage of susceptibility to fosfomycin is very high (over 80%), but still lower than for *E. coli* strains (above 95%) [20]. Seventeen studies conducted around the world (USA, France, Spain, UK, Greece, Turkey, Korea, Japan, Thailand), which analyzed 784 ESBL-producing *Klebsiella pneumoniae* isolates, reported that as many as 81% of them showed susceptibility to fosfomycin [1]. Relatively similar results were obtained in this study—in the group of strains that produced ESBL but did not produce carbapenemases, the percentage of susceptible strains was 75%.

In 2010–2016, studies were conducted in Germany by Putensen et al. on the use of intravenous fosfomycin in daily clinical practice [43]. The research concerned patients with severe, life-threatening infections caused by problematic strains of gram-positive and gram-negative bacteria, including *Klebsiella pneumoniae.* The organisms were isolated from various clinical sources such as CNS infections, pneumonia, urinary tract infections, sepsis, bone and joint infections, and soft tissue infections. Therapeutic success in the use of fosfomycin was noted for as many as 81% of all infections and for 80% of infections caused by *Enterobacterales*. Fosfomycin was mainly used in combination with, depending on the case, carbapenems or cephalosporins [43].

Similar results, proving the relatively high susceptibility of *Klebsiella* multidrug-resistant strains to fosfomycin, were obtained in the research carried out for the purposes of this paper. Data reliability was verified by comparing the obtained determinations with the reference method (agar dilution), which is recommended by EUCAST (Figure 3). The Phoenix automated system and the E-test diffusion method were the alternative methods for determining susceptibility to fosfomycin (Figure 4). The obtained results show that using the reference method, 65% of *Klebsiella* strains were found to be susceptible to fosfomycin, and 35% of the tested strains developed resistance against this drug (Figure 5), which is unfortunately a significant value. Full agreement in terms of susceptibility categories was obtained with the E-test method, which will be discussed later in the paper. Characteristics are very high minimum inhibitory concentrations of resistant strains in relation to the breakpoint of 32 μg/mL (Supplementary Table S3). However, it should be considered that the tested group included most resistant strains, producing extended-spectrum enzymes and belonging to the so-called “alarm pathogens”. As reported in many publications, in the group of strains not producing ESBL enzymes and carbapenemases, the percentage of strains resistant to fosfomycin is much lower [15,20]. In the case of strains analyzed in this study, the fact that fosfomycin could be applied in the therapy of more than half of the patients is extremely important. An observation that seems particularly interesting is the preservation of susceptibility to fosfomycin in bacteria otherwise resistant to drugs regarded as drugs of last resort such as carbapenems or colistin. Six of the analyzed strains showed susceptibility to fosfomycin, despite the developed resistance to colistin. Fifty-five percent of the tested *Klebsiella pneumoniae* strains categorized as “Resistant” or “Susceptible, increased exposure” to carbapenems were found to be susceptible to fosfomycin, which allows for the application of this drug as a therapeutic option for seriously ill patients.

The literature includes reports on the identification of various mechanisms of resistance to fosfomycin. There are three fundamental mechanisms of antimicrobial resistance: enzymatic degradation or inactivation drugs (enzymatic resistance), alteration of bacterial proteins that are antimicrobial targets (receptor resistance), and limitation of drug uptake or active drug efflux (transport resistance). Amino acid changes at the drug binding site are caused by a mutation in the *murA* gene, while mutations in the *glpT, uhpT, cyaA,* and *ptsI* genes hinder the transport of fosfomycin through the bacterial cell wall. Enzymatic resistance, consisting in the production by gram-negative bacteria of glutathione S-tranferase (FosA), inactivating the tested drug, is relatively rare [40]. Research on ESBL-positive strains of *Klebsiella pneumoniae* conducted in Taiwan is among many supporting this thesis. Among the group of fosfomycin-resistant bacteria, 70% had a MurA amino acid substitution, and 96% of this group had functionless transporters [42]. Studies on the enzymatic mechanism of fosfomycin resistance in *Klebsiella pneumoniae* showed a higher resistance rate in carbapenemase-producing strains than in strains producing extended-spectrum β-lactamases. This phenomenon is related to the transfer of the plasmid carrying *fosA3* genes and the gene responsible for the production of carbapenemases [44]. Given the widespread use of the drug, the method of transmission of this resistance (on movable plasmids encoding carbapenemases) may soon reverse the statistics concerning fosfomycin resistance of *Klebsiella.* Fosfomycin susceptibility analysis conducted for carbapenemase-positive strains performed as part of our research (Figure 6) shows a higher proportion of resistant strains in this group (45%), compared to the group of carbapenemase-negative strains (25%), which may indicate the above-described resistance mechanism. This, of course, requires confirmation in further research of the presence of *fosA3* genes on carbapenemase-encoding plasmids. Such studies concerning carbapenemase-positive *Klebsiella* strains are planned by the authors of this paper.

A separate problem, already indicated above, is the choice of laboratory method for the determination of fosfomycin resistance. Despite EUCAST’s recommendation of agar dilution as the reference method, there is a certain freedom of choice of methods used by microbiological laboratories related, for example, to their use of specific apparatuses, which carries a risk of inconsistency of results between methods. The method’s reliability in determining susceptibility of *Enterobacterales* to the test antibiotic is pivotal to effective therapy. The variety of available methods is the subject of many studies [20,21,35,36,39,40,45,46] aimed at finding a test that produces reliable results and at the same time is easy to perform and cost-effective. The extensive literature cited in this paper includes the results of studies comparing the assessment susceptibility to fosfomycin for *Enterobacterales* (including *Klebsiella pneumoniae*), obtained by the reference method and by alternative methods. All experiments lead to the same conclusion—the lack of satisfactory concordance of results of tests carried out using different methods. The studies compare diffusion methods (strips with a gradient of drug concentrations and antibiotic-soaked discs) and broth dilution methods (automated) with the reference method—dilution of the drug in a solid medium. Regarding all alternative methods, the authors [20,36,40,45] demonstrated their weaker ability to detect resistant isolates, which means lower reliability of the tests. Most of the data analyzed in the literature show similarity of results obtained using diffusion methods and the reference method [21,35,40]. On the other hand, there are publications where the automatic methods of diluting the agent in broth seem most compatible with the reference method [20]. There is speculation that fosfomycin resistance may be falsely underestimated in laboratories using only automated susceptibility testing systems. The process of drug diffusion in agar occurring in both diffusion methods and in the reference method (agar dilution) can be the key factor producing similar results of these tests [40,45]. On the other hand, there are also isolated reports suggesting a greater analogy between automated systems and the reference method. A possible reason for these discrepancies may be small test groups or using reagents with different characteristics [20]. Ballestero-Tellez et al. presented the effect of bacterial inoculum on the minimum concentration inhibiting the growth of microbes. The results of fosfomycin agar dilution and other methods were shown to be more similar when using similar bacterial density. This phenomenon may be one of the reasons for the lack of congruence between methods [46].

The results of the determination of *Klebsiella pneumoniae* susceptibility to fosfomycin presented in this paper (Table 1, Figure 5, Figure 6 and Figure 7) support the thesis that the results obtained by the E-test are the most in line with the reference method, as opposed to the Phoenix automated dilution method, in which the MIC values are significantly understated (Figure 8, Supplementary Figure S4). The E-test method was found to be in 100% categorical agreement with the reference method, and no major errors or very major errors (ME and VME) were found, while their percentage in the Phoenix method was 17% and 33%, respectively (Table 1). Unfortunately, the Phoenix method produced as much as 11.6% of results identified as falsely susceptible and 4.7% as falsely resistant (Figure 7B), and no such results were obtained using the E-test method (Figure 7A).

As compared to the reference method, differences in MIC values concern 74% of strains in the E-test method and as many as 86% in the automated method (Figure 9). However, the discrepancy in MIC values varies from small to spectacular differences in drug concentration. Importantly, in the E-test method, as many as 72% of discrepancies are minor differences, and 26% of the results are in complete agreement with the reference method (Figure 9). As already mentioned, there is also no result that would put a strain into a different susceptibility category, which prevents incorrect treatment. This is not the case with the Phoenix automated system, where as many as 35% of results show very large discrepancies in the MIC values compared to the reference method. Of particular concern is the fact that as much as 16% of the strains (seven strains) were assigned to different categories (Susceptible, Resistant) (Figure 9). In such case, a strain is usually categorized as Susceptible, although the reference method indicates Resistant. This creates a high risk of therapeutic failure. Therefore, the Phoenix automated system seems to be the least reliable in comparison with the reference method.

Despite the reference status, the agar dilution method is not used in the routine work of hospital laboratories to determine bacterial susceptibility to fosfomycin. The tedious and time-consuming procedure of preparing suitable media limit large-scale use of the tests. The unquestioned advantages of this method, such as unambiguous, easily readable results (Figure 3) and the high repeatability of results [20], are overshadowed by a number of disadvantages accompanying its performance (e.g., the possibility of performing inaccurate dilutions of the drug, inactivation of the antibiotic at too high agar temperature). The problem could be solved by easily available, easy-to-use, certified commercial tests such as AD Fosfomycin 0.25–256 (Liofilchem, Waltham, MA, USA), but their very high price is an obstacle [39]. In the presented research, neither of the two assessed methods produces results that are completely in line with the reference method. A potential problem with the E-test method may be a certain subjectivity of the reading (heteroresistance phenomenon), given EUCAST recommendations for ignoring single colonies in the growth inhibition zone (Figure 4). Because of large discrepancies in the quoted publications, further research is required to analyze the effectiveness of other methods. Despite the small test group, our results confirmed the thesis that, in accordance with EUCAST recommendations, to determine fosfomycin susceptibility correctly, diffusion and automated methods should be replaced with the reference method, which is undoubtedly facilitated by the emergence of commercial tests eliminating the labor-intensiveness of this method.

In light of its proven activity against *Klebsiella* strains, a high safety profile, and its small molecules easily penetrating into hard-to-reach places in the organism, fosfomycin is a plausible option for treating severe systemic infections caused by multidrug-resistant bacilli. However, due to the increase in the use of fosfomycin—although no such trend has been confirmed in the present paper (Figure 2)—further research in this regard will be necessary [20]. As oral fosfomycin (fosfomycin trometamol) was used for more than 40 years, mainly for the treatment of uncomplicated urinary tract infections, in this study, we have tested strain susceptibility to fosfomycin depending on the site of infection. There is a danger that strains isolated from urine may show higher resistance to this drug. However, no statistically significant correlation was found between the strain isolation site and its fosfomycin susceptibility (Table 2). This may be due to the small number of individual groups, which prompted the authors to plan further research in the following years. Additionally, in the case of infections caused by carbapenemase-producing *K. pneumoniae* strains, fosfomycin should be used judiciously and only in the absence of other therapeutic options. This concern in particular is a concern when a spread of fosfomycin-resistant clones is observed [20,44].

## 4. Materials and Methods

### 4.1. Bacterial Strains

The study used 43 multidrug-resistant *Klebsiella pneumoniae* strains from the collection of the Department of Pharmaceutical Microbiology and Parasitology, Wroclaw Medical University in Poland. These strains were isolated in the last ten years, from the beginning of 2011 to March 2020, from in-patients of four multi-specialty hospitals: Lower Silesian Specialist Hospital in Wroclaw (*n* = 24), Teaching Hospital in Wroclaw (*n* = 14), Regional Specialist Hospital in Wroclaw (*n* = 3), and Regional Specialist Hospital in Legnica (*n* = 2). The organisms were collected from 43 patients showing clinical signs of nosocomial infections and from hospitalized asymptomatic carriers. *Klebsiella* strains were isolated from urine samples (*n* = 15), bronchial aspirates (*n* = 9), other clinical materials (blood, cerebrospinal fluid, pus) (*n* = 7), and from the fecal matter of carriers (*n* = 12) (Supplementary Table S1). Only multidrug-resistant *Klebsiella* strains were selected for testing. All of them had ESBL enzymes, and 20 strains also showed the presence of carbapenemases. 

### 4.2. Microbiological Assays

#### 4.2.1. Automated System

Identification of strains and determination of drug sensitivity and resistance mechanisms were performed using an automated system—Phoenix 100™ (Becton Dickinson, Sparks, MD, USA) with ID402 panels (Becton Dickinson, Sparks, MD, USA) containing a set of 20 antibiotics (amikacin, gentamycin, tobramycin, ertapenem, imipenem, meropenem, cefuroxime, ceftazidime, cefotaxime, cefepime, ampicillin, piperacillin, amoxicillin/clavulanic acid, piperacillin/tazobactam, colistin, cotrimoxazole, fosfomycin, ciprofloxacin, levofloxacin, tigecycline) based on the method of serial dilutions in a liquid medium.

#### 4.2.2. Gradient Diffusion Method (E-Test)

MIC determinations for fosfomycin were also made using the gradient method (E-test) Fosfomycin FM 0.064–1024 μg/mL (bioMerieux Poland, Warsaw, Poland) according to EUCAST guidelines (S ≤ 32, R > 32) for all isolates [47]. The plates were incubated at 35 ± 2 °C for 16–20 h. According to the guidelines, single colonies in the growth inhibition zone were not taken into account.

#### 4.2.3. Reference Method

The dilution method in the Mueller–Hinton agar (MHA) (bioMerieux Poland, Warsaw, Poland) was used as the reference test. This method is the only one recognized by the American Clinical and Laboratory Standards Institute (CLSI) for the purposes of determination of susceptibility to fosfomycin, and is the one most recommended by the EUCAST [48,49,50]. The reference method media were prepared in the laboratory from MHA (dry mass) with an addition of 25 μg/mL of glucose-6-phosphate. The antibiotic media in the MIC ranges of 1–512 μg/mL were prepared according to the EUCAST Definitive Document E. Def 3.1 2000 [19] using a water bath with a temperature of 40 °C and subsequent dilutions of fosfomycin added to liquid agar (Sigma-Aldrich, Saint Louis, MO, USA). A bacterial inoculum with a density of 0.5 McFarland standard was diluted in saline at a ratio of 1:10. Two milliliters of the suspension of the test strains were spotted on the MHA plates with the appropriate antibiotic dilutions. The plates were incubated at 35 ± 2 °C for 16–20 h. The lowest concentration of the antibiotic in the agar, which completely inhibited bacterial growth, was regarded as the MIC for fosfomycin [19]. The results were interpreted in accordance with EUCAST criteria (S ≤ 32, R > 32) [48].

#### 4.2.4. Carbapenemase Identification

Various β-lactamases of Klebsiella bacilli were screened using combination discs (Becton Dickinson, Sparks, MD, USA). ESBL enzymes were identified with DDT (Double-disk test) using combination discs (Becton Dickinson, Sparks, MD, USA) with cefotaxime (30 µg), ceftazidime (30 µg), cefepime (30 µg), and amoxicillin with clavulanic acid (30 µg). MBL carbapenemases were identified using DDST (double-disk synergy test) with discs with imipenem (10 µg), ceftazidime (30 µg), and EDTA (Standard). KPC enzymes were identified using a review method using the following discs: meropeneme 10 µg and meropeneme 10 µg with 10 µL boronic acid (GRASO BIOTECH, Starogard Gdański, Poland) [47,51]. Identification of individual carbapenemases was confirmed using the commercial test Coris Bioconcept RESIST-4 O.K.N.V. (immunochromatographic lateral flow assay for the rapid detection of OXA-48, KPC, NDM, and VIM carbapenemases from cultured isolates) (Argenta, Poznań, Poland) [52].

Quality control for all methods was performed using the following reference strains: *Escherichia coli* ATCC 25922 (MIC range 0.5–2 μg/mL) and *Pseudomonas aeruginosa* ATCC 27853 (MIC range 2–8 μg/mL). All identifications were performed in triplicate [53].

### 4.3. Statistical Analysis

Statistical analysis was performed using TIBCO Software Inc. (2017) Statistica (data analysis software system), version 13, https://www.statsoft.pl/ (accessed on 24 March 2021). Categorical agreement was calculated, and discrepancies were described as very major error (VME; reference method resistant, test method susceptible) and major error (ME; reference method susceptible, test method resistant). The VME rate was calculated by dividing the number of errors (VME) by the number of all resistant isolates. In the case of susceptible isolates, the ME rate was calculated analogically. Fisher’s exact test was used to compare the relationships between categories. Pearson’s correlation coefficient was used to compare the MIC values obtained by the three methods. Due to limitations of the methods, it was not possible to determine the MIC in an identical concentration range. For the purposes of correlation analysis, the values indicated by the inequality sign were replaced with integers. A replacement of value does not affect the diagnostic decision. For “greater than” values, the nearest MIC value on the logarithmic scale was assumed (values >512 were replaced with 1024, and values >64 were replaced with 128). Values "less than or equal to" were replaced with "equal to" values (≤16 was assumed as 16). For all statistical analyses, the *p*-value of <0.05 was adopted as significant. The charts were made using Microsoft Excel, Microsoft 365, https://office.microsoft.com/excel (accessed on 22 March 2021).

## 5. Conclusions

Fosfomycin appears to be the antibiotic with a potential for use in the treatment of infections with multidrug-resistant *Klebsiella* strains, as over 65% of strains are susceptible to this drug. Some strains resistant to colistin and carbapenems are also susceptible, although the percentage of resistance in carbapenemase-producing strains is higher. The E-test can be an alternative to the reference method in routine determination of fosfomycin susceptibility, as it demonstrates agreement in terms of sensitivity categories and, for the most part, only slight differences in MIC values. Subjectivity of the reading is its disadvantage. On the other hand, the serial dilution method employed in the automated Phoenix system seems to be of little use for determining susceptibility to this antibiotic. In comparison to the reference method, it shows large discrepancies in the MIC values and in the susceptibility category.

## Figures and Tables

**Table 1:** Categorical agreement and error rates for two methods vs. the reference method.

Method	Number of Isolates	Categorical Agreement %	ME Rate %	VME Rate %
E-test	43	100 (43/43)	0	0
Phoenix	43	86 (37/43)	17 (2/43)	33 (4/43)

**Table 2:** Susceptibility to fosfomycin by site of isolation.

Isolation Site	Susceptible to Fosfomycin	Resistant to Fosfomycin	Number of Isolates
Urine	9 (20.9%)	6 (13.95%)	15
Faecal matter	6 (13.95%)	6 (13.95%)	12
Bronchial aspirate	7 (16.28%)	1 (2.32%)	8
Blood	3 (6.98%)	0	3
Fistula	1 (2.32%)	1 (2.32%)	2
Gastric washing	0	1 (2.32%)	1
CSF	1 (2.32%)	0	1
Sputum	1 (2.32%)	-	1

## Data Availability

All data are provided in the main body and Supplementary Data of the manuscript.

## Supplementary Materials

The following are available online at https://www.mdpi.com/article/10.3390/pathogens10050512/s1, Table S1: List of *Klebsiella pneumoniae* strains isolated in different hospital centers, Table S2: Results of determination of resistance mechanisms in *Klebsiella* strains, Table S3: The results of *Klebsiella* fosfomycin susceptibility study with the MIC value, determined by different methods, Figure S1: MIC distribution of fosfomycin for tested *Klebsiella pneumoniae* strains.
