# Supplementary material for: In Vitro Susceptibility of Multi-Drug Resistant Klebsiella pneumoniae Strains Causing Nosocomial Infections to Fosfomycin. A Comparison of Determination Methods
Source: Pathogens. 2021 Apr 23;10(5):512. doi: 10.3390/pathogens10050512 (PMC8145326; doi:10.3390/pathogens10050512)
Supplement: Supplementary file 1 [file pathogens-10-00512-s001.zip › pathogens-1160284-supplementary.pdf]

Table S1. List of *Klebsiella pneumoniae* strains isolated in different hospital centers.

Strain number	Initials	Sex	Material	Date of isolation	Branch
**A. Isolated strains in Lower Silesian Specialist Hospital in Wroclaw**					
1	M.S.	M	Urine	22.09.2019	Orthopaedic Trauma Surgery Department
2	K.K.	F	Urine	20.09.2019	Department of Cardiology
3	M.W-S.	F	Bronchial aspirate	22.09.2019	Department of Toxicology and Internal Diseases
4	W.T.	F	Urine	28.09.2019	Department of Urology and Oncological Urology
5	J.W.	M	Urine	29.09.2019	Department of Anaesthesiology and Intensive Care
6	M.R.	F	Urine	01.10.2019	Department of Internal Diseases
7	B.W-P.	M	Urine	01.10.2019	Department of Neurology with Stroke Subdivision
8	B.P.	M	Bronchial aspirate	09.10.2019	Department of Neurology with Stroke Subdivision
9	M.J.	F	Bronchial aspirate	11.10.2019	Department of Anaesthesiology and Intensive Care
10	J.K.	F	Urine	31.03.2017	Orthopaedic Trauma Surgery Department
11	S. J	M	Bronchial aspirate	10.05.2017	Department of Anaesthesiology and Intensive Care
12	A.S.	M	Bronchial aspirate	19.05.2017	Department of Anaesthesiology and Intensive Care
13	B.W-L.	F	CSF	14.08.2017	Department of Neurology with Stroke Subdivision
14	R.A	F	Urine	12.01.2018	Department of Internal Diseases
15	I. D	F	Urine	21.01.2018	Department of Internal Diseases
16	R.W.	F	Feces (carrier)	16.01.2018	Department of Internal Diseases
17	W.P.	M	Urine	09.11.2019	Department of Neurochirurgii
18	I.U.	M	Bronchial aspirate	09.11.2019	Department of Anaesthesiology and Intensive Care
19	T.K.	M	Urine	31.10.2019	Orthopaedic Trauma Surgery Department
20	G.D	F	Urine	16.11.2019	Department of Cardiology
21	M.M	F	Blood	04.11.2019	Department of Neurology with Stroke Subdivision
22	A.K.	F	Blood	22.02.2020	Department of Anaesthesiology and Intensive Care
23	B.M.	F	Bronchial aspirate	28.02.2020	Department of Anaesthesiology and Intensive Care
24	I.K.	F	Bronchial aspirate	04.03.2020	Department of Anaesthesiology and Intensive Care
**B. Isolated strains in the Regional Specialist Hospital in Wroclaw**					
**Strain number**	**Initials**	**Sex**	**Material**	**Date of isolation**	**Branch**
25	W.M.	M	Abdominal flushes	11.2017	Department of Infectious Diseases
26	S.G.	M	Feces (carrier)	11.2017	Department of Infectious Diseases
27	M.W.	F	Feces (carrier)	11.2017	Department of Infectious Diseases
**C. Isolated strains in the Regional Specialist Hospital in Legnica**					
**Strain number**	**Initials**	**Sex**	**Material**	**Date of isolation**	**Branch**
28	Z.B.	F	Purulent mite swab (wound)	11.2017	Haematological Department
29	S.K.	M	Feces (carrier)	12.2017	Department of InternalDiseases
**D. Isolated strains at the University Clinical Hospital in Wroclaw**					
**Strain number**	**Initials**	**Sex**	**Material**	**Date of isolation**	**Branch**
30	M.W.	M	Urine	18.10.2017	Clinic of Hematology, Blood Cancers and Bone Marrow Transplantation
31	P. S.	M	Blood	25.05.2014	Clinic of Hematology, Blood Cancers and Bone Marrow Transplantation
32	K.K.	M	Feces (carrier)	30.01.2015	Clinic of Hematology, Blood Cancers and Bone Marrow Transplantation
33	B.A.	F	Feces (carrier)	17.11.2014	Clinic of Hematology, Blood Cancers and Bone Marrow Transplantation
34	K.w.	M	Feces (carrier)	20.05.2014	Clinic of Hematology, Blood Cancers and Bone Marrow Transplantation
35	W.M.	M	Urine	03.11.2011	Clinic of Hematology, Blood Cancers and Bone Marrow Transplantation
36	M.U.	F	Feces (carrier)	05.06.2014	Clinic of Hematology, Blood Cancers and Bone Marrow Transplantation
37	L.T.	M	Feces (carrier)	11.06.2013	Clinic of Hematology, Blood Cancers and Bone Marrow Transplantation
38	P.M.	M	Feces (carrier)	12.10.2013	Clinic of Hematology, Blood Cancers and Bone Marrow Transplantation
39	A.J.	F	Spit	16.01.2014	Clinic of Hematology, Blood Cancers and Bone Marrow Transplantation
40	D.A.	F	Feces (carrier)	27.02.2014	Clinic of Hematology, Blood Cancers and Bone Marrow Transplantation
41	Z.B.	M	Purulent mite swab (wound)	13.05.2019	Clinic of General Surgery and Oncological Surgery
42	S.M.	M	Urine	09.05.2017	Department of Anaesthesiology and Intensive Care
43	P.Z.	F	Feces (carrier)	21.09.2016	Clinic of Hematology, Blood Cancers and Bone Marrow Transplantation

Table S2. Results of determination of resistance mechanisms in *Klebsiella* strains.

Strain number	ESBL	MBL	KPC	Susceptibility to fosfomycin in reference methods
**1A**	+	-	-	R
**2A**	+	-	-	S
**3A**	+	-	-	S
**4A**	+	-	-	R
**5A**	+	-	-	S
**6A**	+	-	-	R
**7A**	+	-	-	S
**8A**	+	+	-	R
**9A**	+	-	-	S
**10A**	+	-	-	S
**11A**	+	-	-	S
**12A**	+	-	-	S
**13A**	+	-	-	S
**14A**	+	+	-	S
**15A**	+	+	-	R
**16A**	+	+	-	S
**17A**	+	+	-	S
**18A**	+	-	-	S
**19A**	+	-	-	S
**20A**	+	+	-	S
**21A**	+	-	-	S
**22A**	+	+	-	S
**23A**	+	+	-	S
**24A**	+	+	-	S
**25B**	+	+	-	R
**26B**	+	+	-	R
**27B**	+	+	-	R
**28C**	+	+	-	S
**29C**	+	+	-	S
**20%**	+	-	+	R
**31D**	+	-	-	S
**32D**	+	-	-	R
**33D**	+	-	+	R
**34D**	+	+	-	S
**35D**	+	-	+	R
**36D**	+	-	-	S
**37D**	+	-	+	R
**38D**	+	-	-	R
**39D**	+	-	-	S
**40D**	+	-	-	S
**41D**	+	-	-	R
**42D**	+	-	-	S
**43D**	+	+	-	S

Table S3. The results of *Klebsiella* fosfomycin susceptibility study with the MIC value, determined by different methods.

**Strain Number**	**1A**	**2A**	**3A**	**4A**	**5A**	**6A**	**7A**	**8A**	**9A**	**10A**	**11A**	**12A**	**13A**	**14A**
**Reference Method**	**R**	**S**	**S**	**R**	**S**	**R**	**S**	**R**	**S**	**S**	**S**	**S**	**S**	**S**
	128	32	32	128	8	256	16	>512	16	32	32	32	32	4
**E-test**	**R**	**S**	**S**	**R**	**S**	**R**	**S**	**R**	**S**	**S**	**S**	**S**	**S**	**S**
	96	32	24	64	3	256	6	1064	6	24	32	32	24	16
**Phoenix**	**S**	**S**	**S**	**S**	**S**	**S**	**S**	**R**	**S**	**S**	**S**	**S**	**S**	**R**
	≤16	≤16	≤16	≤16	≤16	≤16	≤16	>64	≤16	≤16	≤16	≤16	≤16	>64

**Strain Number**	**15A**	**16A**	**17A**	**18A**	**19A**	**20A**	**21A**	**22A**	**23A**	**24A**	**25B**	**26B**	**27B**	**28C**
**Reference Method**	**R**	**S**	**S**	**S**	**S**	**S**	**S**	**S**	**S**	**S**	**R**	**R**	**R**	**S**
	128	32	16	32	32	32	32	1	32	32	128	64	128	32
**E-test**	**R**	**S**	**S**	**S**	**S**	**S**	**S**	**S**	**S**	**S**	**R**	**R**	**R**	**S**
	96	24	6	24	16	24	24	0,064	12	24	64	64	64	16
**Phoenix**	**R**	**S**	**S**	**S**	**S**	**S**	**S**	**S**	**S**	**S**	**S**	**R**	**S**	**S**
	>64	≤16	≤16	≤16	≤16	≤16	≤16	16	≤16	≤16	≤16	64	≤16	≤16

**Strain Number**	**29C**	**20%**	**31D**	**32D**	**33D**	**34D**	**35D**	**36D**	**37D**	**38D**	**39D**	**40D**	**41D**	**42D**	**43D**
**Reference Method**	**S**	**R**	**S**	**R**	**R**	**S**	**R**	**S**	**R**	**R**	**S**	**S**	**R**	**S**	**S**
	32	>512	32	256	128	32	>512	32	512	256	32	32	256	32	32
**E-test**	**S**	**R**	**S**	**R**	**R**	**S**	**R**	**S**	**R**	**R**	**S**	**S**	**R**	**S**	**S**
	16	256	16	256	64	12	1064	24	256	192	32	24	256	12	16
**Phoenix**	**S**	**R**	**S**	**R**	**R**	**S**	**R**	**R**	**R**	**R**	**S**	**S**	**R**	**S**	**S**
	32	64	≤16	>64	64	≤16	>64	>64	>64	64	32	≤16	64	≤16	≤16

S—susceptible, **R**—resistant, numerical values—MIC in μg/mL.

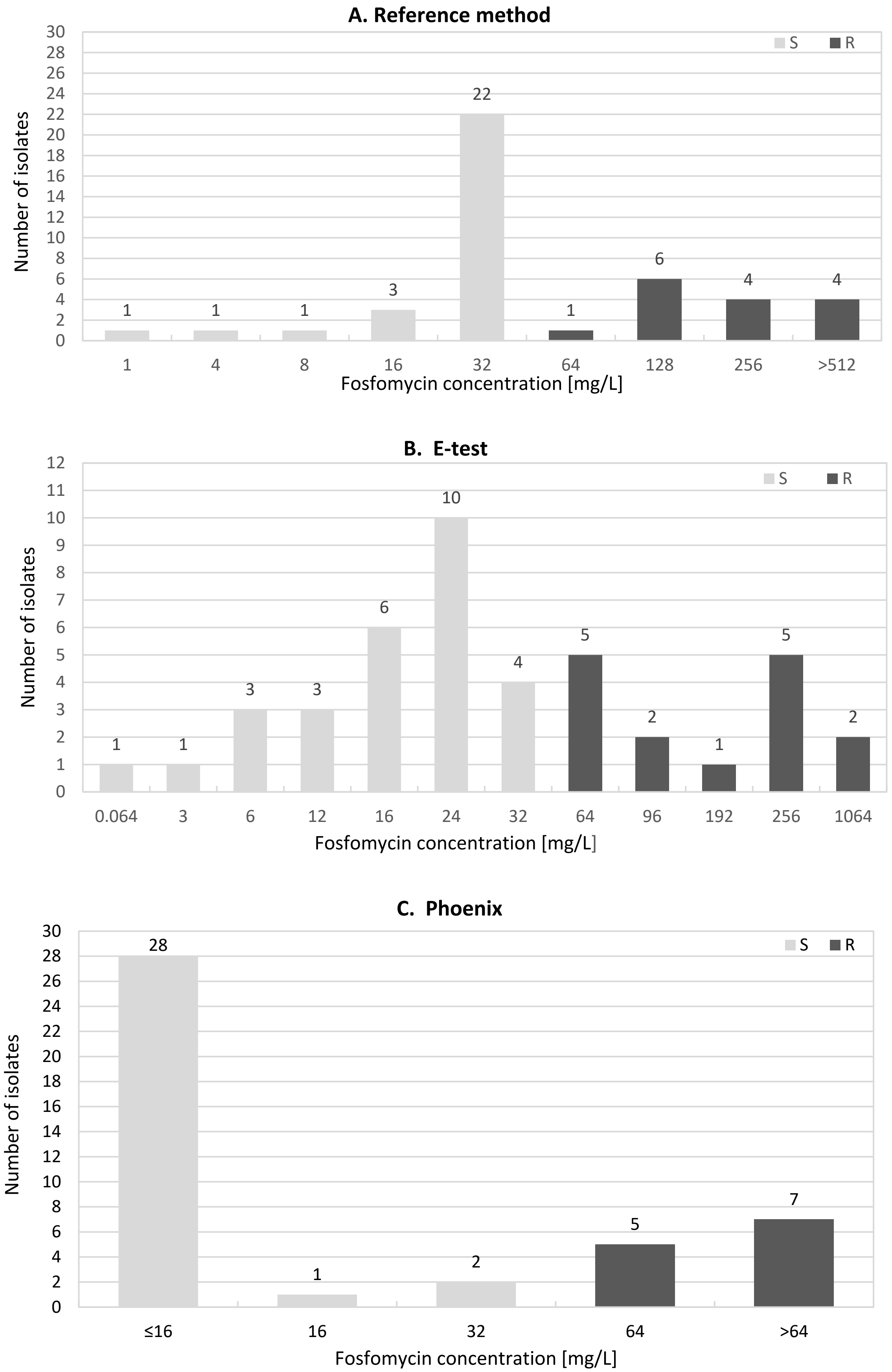

Figure S1. MIC distribution of fosfomycin for tested *Klebsiella pneumoniae* strains; S—susceptible, R—resistant in different methods: (**A**) Reference method; (**B**) E-test; (**C**) Phoenix.
